# Molecular profiling of breast cancer methylation pattern in triple negative versus non- triple negative breast cancer

**DOI:** 10.1038/s41598-025-90150-9

**Published:** 2025-02-26

**Authors:** Marwa Mohanad, Hager M. Hamza, Abeer A. Bahnassy, Sabry Shaarawy, Ola Ahmed, Hatem A. EL-Mezayen, Eman G. Ayad, Neveen Tahoun, Mona S. Abdellateif

**Affiliations:** 1 Biochemistry Department, College of Pharmaceutical Sciences and Drug Manufacturing, Misr University for Science and Technology, 6th October, Egypt; 2 Chemistry Department, Faculty of Science, Helwan University, Cairo, Egypt; 3 Pathology Department, National Cancer Institute, Cairo University, Cairo, Egypt; 4 Medical Biochemistry and Molecular Biology, Cancer Biology Department, National Cancer Institute, Cairo University, Cairo, Egypt

**Keywords:** Breast cancer, Methylation, TNBC, *CCND2*, *PTEN*, *BRCA1*, *TNFRSF10C*, *GSTP*, Cancer, Molecular biology, Biomarkers

## Abstract

Epigenetic alterations, especially promotor methylation, have a significant impact on gene expression, molecular subtyping, prognosis, and outcome of breast cancer (BC). The methylation profile was assessed for 22 genes of the BC tissue using the EpiTect Methyl II PCR System in 40 triple-negative BC (TNBC) patients compared to 50 non-TNBC group. The data were corelated with the disease-free (DFS) and overall survival (OS) of the patients. Genes that were differentially hypermethylated in TNBC patients compared to those with non-TNBC included *CCND2, CDKN2A, ESR1, CDH1, BRCA1, GSTP, RASSF1, SLIT2, MGMT, PTEN, TP73*, and *PRDM2*. These panel achieved 95% sensitivity, 98% specificity, 97.44% positive predictive value (PPV), 94.23% negative predictive value (NPV), and AUC of 0.993. Hypermethylation of *BRCA1, CDH1, CDKN2A, ESR1, GSTP, HIC1, MGMT, PRDM2, PTEN, PYCARDM, RASSF1M, THBS1*, and *TP73* associated significantly with worse OS and DFS in TNBC cohort. Meanwhile, *CCNA1* and *CDH1* hypermethylation demonstrated significant associations with poor DFS but did not show significant relationships with OS in TNBC patients. *PTGS2* and *TNFRSF10C* methylation were associated with better DFS and OS rates in TNBC patients. On multivariate Cox regression, *CCND2* and *PTEN* hypermethylation were independent predictors of DFS in the overall BC patients. The hypermethylation of *BRCA1* and *GSTP* were independent predictors of DFS, while *PTEN* hypermethylation was an independent predictor of OS in the TNBC cohort. The identification of hypermethylated genes, such as *BRCA1, CCND2, CDH1, ESR1, GSTP, RASSF1, SLIT2, MGMT,* and *PTEN* may serve as potential biomarkers or therapeutic targets for TNBC.

## Introduction

Female breast cancer (BC) is the first cause of global cancer in incidence in 2020 representing 11.7% of all cancer cases, and it ranks the fifth leading cause of cancer deaths worldwide^1^. It was reported that BC accounted for 685,000 women deaths, which represents 16% of women cancer deaths^1,2^. Despite a better understanding of the several etiological factors for BC, its incidence and mortality rates continue to increase steadily^3^. This is due to the fact that BC is a heterogenous disease with variable pathological, molecular, and biological subtypes that influence the prognosis and management of BC patients^4^.

Currently, there is a well-defined targeted therapy for the non-triple negative BC (TNBC) subtypes which are positive for estrogen (ER), progesterone (PR), and/or epidermal growth factor receptor 2 (HER2) receptor expression^4^. The other TNBC subtype, which accounts for about 15%-20% of BC cases, and characterized by the absence of ER, PR, and HER2 expression^5^. Recent research demonstrated that TNBC is a heterogenous group of tumors with different underlying molecular aberrations^6,7^. It is usually presented in young females with an advanced disease stage, high histological grade, reduced survival rate, and increased incidence of relapse, that makes it the most aggressive subtype of BC^5,8^.

In fact, the treatment of TNBC is a great challenge due to the absence of hormonal and HER2 receptor expression. Consequently, neoadjuvant or adjuvant cytotoxic chemotherapy remains the only available treatment for those patients^5,9^. Though there was a high response rate, especially in early-stage TNBC patients, there was an increased incidence of recurrence and drug resistance, which ultimately led to a poor survival rate^10^.

Epigenetic alterations particularly promotor hypermethylation are known to have an important role in the initiation and progression of many cancers including BC^11–13^. DNA methylation is the addition of a methyl group to the cytosine residue predominantly at the CpG island. This process occurs by the DNA methyltransferase enzymes (DNMT1, DNMT3A, DNMT3B), which transfer the methyl group from the S-adenosyl-L-methionine to the DNA cytosine ring^14^. It had been found that DNA methylation patterns associated significantly with the clinicopathological features of BC patients including tumor grade, stage, and TP53 status^15,16^. The DNA hypermethylation or hypomethylation can affect the pathogenesis and the progression of BC through downregulation of tumor suppressor genes or activation of oncogenes; respectively^17^. Noteworthy, promotor hypermethylation profile was reported to vary according to the BC subtypes, e.g. the ER1/luminal BCs are associated with a higher frequency of DNA methylation compared to ER2/basal-like tumors^18^. Therefore, several DNA-methylated genes were assessed as molecular biomarkers for BC, and they were reported to have considerable diagnostic and prognostic significance in BC patients^19–22^.

In the current study, we tried to identify a DNA methylation profile including 22 genes in TNBC compared to non-TNBC. This will allow us to find a panel of a DNA methylation signature that could have a diagnostic, prognostic, or predictive role in TNBC patients, aiming to be a promising targeted therapy for patients with this type of BC.

## Patients and methods

This is a retrospective cohort study included 90 Egyptian females with histopathologically confirmed BC. Patients were diagnosed and treated at the National Cancer Institute (NCI), Cairo University during the period from 2011 to 2019 according to the standard protocols of the NCI. Patients included in the study were egyptian BC patients who had no history of other malignancy, had no history of previous chemotherapy in adjuvant or metastatic settings, had no severe uncontrolled concomitant disease, or inadequate organ function that would contraindicate the use of the drugs. All patients were subjected to full history taking, full clinical examination, complete laboratory, and radiological assessment. Patients were also assessed for any adverse events as well as tumor response after 4, 8, 16 and 24 weeks of treatment. The management of the patients was performed acording to the NCI guidelines, where surgical resection is the primery treatment for eligible cases, followed by anthracycline-based therapy. Taxanes was aded to high-risk patients including HER2 overexpression, in those patients adjuvant Trastuzumab usually considered for 1 year. In patients with hormone positive BC, adjuvant hormonal treatment was adminsterated for 5 years either Tamoxifen for premenopausal females, and Tamoxifen/aromatase inhibitors (AI) in high-risk post menopausal patients. Patients with metastatic BC were treated with hormonal therapy if hormone-positive low volume disaese. While those with hormone negative and/or large volume disease were treated with anthracyclin based combination chemotherapy in front-line setting (if not previously admnisterated). Other options includes Taxanes, Capecitabine, Gemcitabine, Vinorolbine, Platinum salts and other drugs usually as single agent.

### Samples collection

The representative paraffin blocks for each case were identified and assessed for the ratio of tumor to normal cells, where samples were included if it had ≥ 75% neoplastic cells. Five sections (5 µm each) were taken from each tumor block and stored in a sterile 1.5 mL Eppendorf tube for DNA extraction and methylation assessment.

### DNA extraction

DNA was extracted from the representative sections of BC cases using QIAamp DNA FFPE tissue kit (Qiagen, Germany, Cat. No. 56404) according to manufacturer’s protocol. The extracted DNA was assessed for the concentration and purity using spectrophotometer NanoDrop (Quawell, Q-500, Scribner, USA).

### Methylation specific PCR

The methylation profile was assessed for 22 genes of the BC tissue using the EpiTect Methyl II PCR System (Quiagen, Meryland, USA, Cat. No. 335212). This technology provides ready-to-use, predesigned primers to detect the methylation status of the promoter region (gene) of interest with high specificity and amplification efficiency. This system was chosen due to its ability to quantitatively measure DNA methylation without requiring bisulfite conversion, which can cause DNA degradation and loss. By combining methylation-sensitive and methylation-dependent restriction enzymes, this method provides high sensitivity and specificity in detecting methylated and unmethylated DNA regions. The system is optimized for analyzing DNA extracted from formalin-fixed paraffin-embedded tissues, making it ideal for clinical and archival samples. Moreover, it requires less DNA input (as low as 20 ng per reaction) and is compatible with any real-time PCR instrument, making it a robust and accessible tool for methylation profiling. The assay was detected with the MethylScreen® technology provided under license from Orion Genomics, LLC^23^.

Briefly, the genomic DNA was divided into four equal portions and incubated with restriction endonuclease including the methylation-sensitive and a methylation-dependent restriction enzyme. The four tubes were as follow; the mock (no enzyme), methylation-sensitive (MSRE), methylation-dependent (MDRE), and double (MSRE and MDRE) restriction endonuclease digestion. After digestion, the enzyme reactions were mixed with qPCR master mix in the PCR Array plate. The product of the mock digestion represents the total amount of input DNA for real-time PCR detection. In the methylation-sensitive digestion (Ms) reaction, the MSRE will digest unmethylated DNA, while in the methylation-dependent digestion (Md) reaction, the MDRE will digest methylated DNA. In the double digestion (Msd) reaction, both enzymes are present, and all DNA molecules (both methylated and unmethylated) will be digested. Then the remaining unmethylated DNA will be detected by real-time PCR.

The PCR reactions were performed in the thermal cycler Agilent technologies stratagene (MXx3000p© Agilent Technologies, Inc. Germany) according to the manufacture protocol. The raw ΔCT values are pasted into the data analysis spreadsheet, which automatically calculates the relative amount of methylated and unmethylated DNA fractions. The methylated fraction was calculated as percentage unmethylated (UM) and percentage methylated (M) fraction of input DNA.

### Statistical analysis

All statistical analyses were conducted using R Studio version 4.3.1. Data visualization and clustering were carried out using heatmaps and principal component analysis (PCA). The heatmap was employed to display gene methylation patterns across samples, with hierarchical clustering of genes and samples to uncover distinct clusters. PCA was performed to reduce dimensionality and visualize the separation between TNBC and non-TNBC samples, with biplots illustrating the PCA results. Differences in methylation levels between TNBC and non-TNBC subtypes were assessed using a *t* test. Fold changes indicate the direction and magnitude of differences in methylation between the two groups. A volcano plot was used to highlight the differential methylation between TNBC and non-TNBC samples. This plot facilitated the identification of genes with significant methylation changes, allowing for comparison of effect sizes and statistical significance. The significance level was set at *p* < 0.05.

A Random Forest model was implemented to predict TNBC status and identify key features affecting BC classification using the random forest package in R. The model was optimized through tenfold cross-validation, which ranked variables based on their importance scores, calculated by mean decrease in accuracy and Gini index. Features with the highest importance scores were considered potential predictors for TNBC. Model performance was evaluated using confusion matrices and ROC curves, with key metrics including Accuracy, Sensitivity, Specificity, and ROC AUC. For survival analysis, Cox proportional hazards regression was used to investigate the association between gene methylation and clinical outcomes, such as Disease-Free Survival (DFS) and Overall Survival (OS). The DFS was the estimated period from the time of primary treatment till that of relapse or progressive disease. While the OS was the period from the date of diagnosis till the date of death. Both univariate and multivariate analyses were conducted to assess the impact of individual and combined methylation markers on survival outcomes.

### Ethics approval and consent to participate

The study protocol was approved by the ethical committee of the NCI, Cairo University, which was in accordance with 2011 declaration of Helsinki (no.CB2401-102-052). Signed informed consents were obtained from all patients prior to enrollment in the study.

### Consent for publication

All participated patients and authors agreed for publication.

## Results

### Patients’ characteristics

The current study included 90 BC females with a mean age of 56.64 ± 12.97 years. Patients were categorized into two groups; the non-TNBC group which included 50 patients with a mean age of 52.82 ± 11.86, and the TNBC group which formed of 40 patients with a mean age of 56.20 ± 11.86. Regarding the non-TNBC patients, ER expression was found in 36 (72%) patients, PR expression in 30 (60%) patients, while HER2 expression was present in 17 (34%) patients. There was a significant difference between the two groups regarding the tumor grade (*P* = 0.025), as grade 3 was mostly found in TNBC [14/40 (35%)], compared to the non-TNBC [7/50 (14.0%)]. Similarly, metaplastic carcinoma was detected only in TNBC patients [7/40 (17.5%)], compared to the non-TNBC [0/50 (0.0%)]. While the other pathological subtypes including DCIS, IDC, and adenocarcinoma were nearly equally distributed between the two groups (*P* = 0.022). Relapses were reported in 15/40 (37.5%) TNBC patients, compared to 7/50 (14.0%) non-TNBC patients (*p* = 0.01). Otherwise, there was no significant difference between the two groups regarding laterality, menopausal status, lymph node metastasis, and distant metastasis (*P* = 0.47, 0.28, and 0.24; respectively, Table 1).

**Table 1:** The clinico-pathological features of the assessed breast cancer patients.

	Total (n = 90)	TNBC (n = 40)	Non-TNBC (n = 50)	*p*-value
Age (years)	56.64 ± 12.94	56.20 ± 11.86	52.82 ± 11.86	0.83
Menopausal status				
Pre-menopause	41 (45.5)	17 (42.5)	24 (48)	0.51
Post-menopause	49 (54.5)	23 (57.5)	26 (52)	
Grade (%)				0.025
2	69 (76.7)	26 (65.0)	43 (86.0)	
3	21 (23.3)	14 (35.0)	7 (14.0)	
Laterality				0.47
Right	39 (43.3)	18 (45.0)	21 (42.0)	
Left	51 (56.7)	22 (55.0)	29 (58.0)	
Pathology				0.022
DCIS	2 (2.2)	1 (2.5)	1 (2.0)	
IDC	74 (82.2)	28 (70.0)	46 (92.0))	
Metaplastic carcinoma	7 (7.8)	7 (17.5)	0 (0.0)	
Invasive mammary carcinoma	6 (6.7)	3 (7.5)	3 (6.0)	
Adenocarcinoma	1 (1.1)	1 (2.5)	0 (0.0)	
LN metastasis				0.28
No	47 (52.2)	19 (47.5)	28 (56.0)	
Yes	43 (47.8)	21 (52.5)	22 (44.0)	
Relapse				0.01
No	68 (75.6)	25 (62.5)	43 (86.0)	
Yes	22 (24.4)	15 (37.5)	7 (14.0)	
Metastasis				0.24
M0	82 (91.1)	35 (87.5)	47 (94.0)	
M1	8 (8.9)	5 (12.5)	3 (6.0)	

### Gene methylation profile between TNBC and non-TNBC patients

**Fig. 1:**
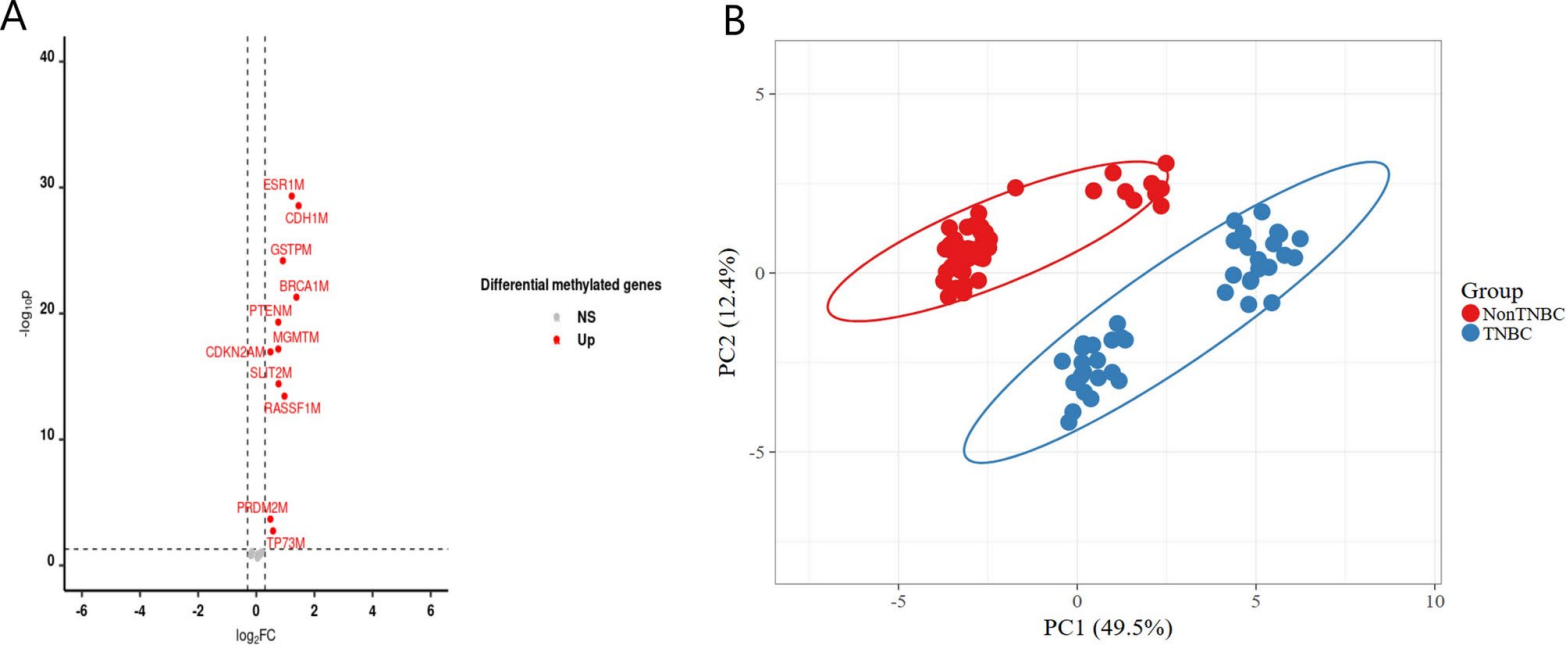
(**A**) Volcano plot showing significant differences between TNBC and non-TNBC patients. (**B**) A principal component analysis (PCA) showing the variability in the methylation status of selected genes in TNBC and non-TNBC samples.

The analysis of gene methylation levels (mean ± standard deviation) revealed significant differences between TNBC and non-TNBC patients, as presented in Table 2 and visualized in the volcano plot (Fig. 1A). Several genes displayed notable variations in their methylation status with statistical significances supported by p-values and fold change (FC) calculations highlighting their potential roles in TNBC pathogenesis. Among the highly hypermethylated genes were those involved in cell cycle regulation and apoptosis such as *CCND2* (FC = 2.80), *RASSF1* (FC = 1.59), and *CDKN2A* (FC = 1.22). Additionally, genes involved in angiogenesis [*CDH1*(FC = 2.74)], tumor suppressor genes [*SLIT2* (FC = 1.48), *PTEN* (FC = 1.47), and *TP73* (FC = 1.21)], DNA repair genes [*BRCA1*(FC = 2.60) and *MGMT* (FC = 1.47)], histone methylation genes [*GSTP* (FC = 1.64), *PRDM2* (FC = 1.06)] and that coded for estrogen receptor *ESR1*(FC = 2.33), suggesting their critical role in the pathogenesis of TNBC.

**Table 2:** Methylation level of genes across TNBC and non-TNBC patients.

	TNBC	Non-TNBC	*p* value	Fold change
*ADAM23* (methylated)	58.3 ± 8.3	56.82 ± 3.62	0.26	1.03
*BRCA1* (methylated)	74.65 ± 16.79	28.70 ± 15.73	5.05E−22	2.60
*CCNA1* (methylated)	54.71 ± 11.89	52.14 ± 5.67	0.17	1.05
*CCND2* (methylated)	85.10 ± 7.72	30.36 ± 5.99	1.31E−48	2.80
*CDH1* (methylated)	81.88 ± 14.07	29.96 ± 14.53	2.76E−29	2.74
*CDH13* (methylated)	71.90 ± 8.96	69.82 ± 6.07	0.194	1.03
*CDKN1C* (methylated)	75.33 ± 8.41	72.58 ± 8.30	0.12	1.04
*CDKN2A* (methylated)	86.00 ± 6.60	70.50 ± 6.94	1.11E−17	1.22
*ESR1*(methylated)	78.08 ± 10.17	33.46 ± 14.15	4.76E−30	2.33
*GSTP* (methylated)	81.20 ± 9.57	49.24 ± 11.41	6.38E−25	1.64
*HIC1* (methylated)	57.23 ± 18.69	50.32 ± 18.199	0.081	1.14
*MGMT* (methylated)	73.80 ± 10.37	50.06 ± 9.77	6.65E−18	1.47
*PRDM2* (methylated)	84.85 ± 6.192	80.06 ± 5.28	0.00021	1.06
*PTEN* (methylated)	80.35 ± 10.42	54.74 ± 8.64	4.84E−20	1.47
*PTGS2* (methylated)	49.05 ± 16.92	54.70 ± 13.30	0.079	0.90
*PYCARD* (methylated)	52.33 ± 15.37	48.34 ± 10.74	0.152	1.08
*RASSF1* (methylated)	75.83 ± 14.77	47.58 ± 14.33	3.63E−14	1.59
*SFN* (methylated)	70.33 ± 7.651	68.04 ± 7.494	0.16	1.03
*SLIT2* (methylated)	74.25 ± 12.88	50.10 ± 6.00	3.80E−15	1.48
*THBS1* (methylated)	51.10 ± 14.77	46.24 ± 14.87	0.126	1.11
*TNFRSF10C* (methylated)	53.98 ± 28.54	61.26 ± 20.478	0.163	0.88
*TP73* (methylated)	66.88 ± 16.70	55.68 ± 14.163	0.0018	1.21

The statistical significance of differences in methylation levels between TNBC and non-TNBC subtypes was assessed using a *t* test (*p* value < 0.05). Fold changes indicate the direction and magnitude of differences in methylation between the two groups.

### Association of gene methylation signature with the DFS in BC, TNBC, and non-TNBC patients

The univariate analysis for the relapse time in all included BC patients showed that worse DFS was significantly observed in patients with hypermethylation of genes involved in cell cycle regulation and apoptosis [*CDKN2A* (HR = 1.19, *p* < 0.001), CCND2 (HR = 1.019, *p* = 0.005), *RASSF1* (HR = 1.10, *p* < 0.001), *TNFRSF10C* (HR = 0.951, *p* < 0.001), *PYCARD*M (HR = 1.169, *p* < 0.001)], genes involved in angiogenesis and metastasis [CDH1 (HR = 1.054, *p* =  < 0.001), *THBS1* (HR = 1.081, *p* < 0.001), *PTGS2* (HR = 0.872, *p* < 0.001)], tumor suppressor genes [*HIC1* (HR = 1.106, *p* < 0.001), *PTEN* (HR = 1.11, *p* < 0.001), *TP73* (HR = 1.079, *p* < 0.001)], DNA repair genes [*BRCA1* (HR = 1.064, *p* < 0.001), *MGMT* (HR = 1.122, *p* < 0.001)], histone methylation genes [*GSTP* (HR = 1.078, *p* < 0.001), *PRDM2* (HR = 1.21, *p* < 0.001)], and that coded for estrogen receptor [*ESR1* (HR = 1.073, *p* < 0.001)].

On the other hand, the methylation of ADAM23, CCNA1, CDH13, CDKN1C, SFN, and SLIT2 did not show any significant associations with DFS, suggesting that these genes have no prognostic value in this cohort.

On multivariate Cox regression, the methylation status of CCND2 [HR = 1.194, *p* < 0.001] and PTEN [HR = 1.190, *p* = 0.012] were independent predictors of DFS in the overall BC cohort (Supp. 1).

In the TNBC subgroup, higher methylation of several genes exhibited a strong association with poor DFS, highlighting its prognostic importance in TNBC. These genes were those involved in cell cycle regulation and apoptosis including *CDKN2A* (HR = 1.28, *p* < 0.001), *CCNA1* (HR = 0.953, *p* = 0.011), *RASSF1* (HR = 1.138, *p* < 0.001), and *PYCARD* (HR = 1.15, *p* < 0.001)]. Similarly, a statistically significant increased risk of worse DFS was associated with hypermethylation of genes involved in angiogenesis and metastasis [*CDH1* (HR = 1.18, *p* < 0.001), *THBS1* (HR = 1.17, *p* < 0.001)], tumor suppressor genes including [*HIC1* (HR = 1.11, *p* < 0.001), *PTEN* (HR = 1.207, *p* < 0.001), *TP73* (HR = 1.089, *p* < 0.001)], DNA repair genes [*BRCA1* (HR = 1.169, *p* < 0.001), *MGMT* (HR = 1.184, *p* < 0.001)], histone methylation genes [*GSTP* (HR = 1.126, *p* < 0.001), *PRDM2* (HR = 1.177, *p* < 0.001)], and that coded for estrogen receptor [*ESR1* (HR = 1.20, *p* < 0.001)].

Conversely, *PTGS*2 [HR = 0.888, *p* < 0.001] and *TNFRSF10C* [HR 0.948, *p* < 0.00] methylation was notably associated with better DFS.

Finally, the methylation of *ADAM23*, *CCND2*, *CDH13*, *CDKN1C*, *SFN*, and *SLIT2* did not show any significant associations with DFS in the TNBC cohort.

Multivariate survival analysis showed that methylation of *BRCA1* [HR = 1.353, *p* = 0.01] and *GSTP* [HR = 1.18, *p* = 0.02] were independent predictors of DFS in the TNBC cohort (Table 3).

**Table 3:** Cox regression analysis for time to progression/relapse (progression-free survival) in overall BC, TNBC and non-TNBC patients.

	Univariate analysis (DFS)			Multivariate analysis (DFS)		
	HR	95% CI	*p* value	HR	95% CI	*p* value
TNBC (n = 40)						
*ADAM23* (methylated)	0.975	0.931–1.022	0.292			
*BRCA1* (methylated)	1.169	1.093–1.250	< 0.001	1.353	1.04–2.015	0.01
*CCNA1* (methylated)	0.953	0.918–0.989	0.011	0.972	0.899–1.051	0.482
*CCND2* (methylated)	1.004	0.952–1.059	0.88			
*CDH1* (methylated)	1.18	1.090–1.284	< 0.001	1.222	0.959–1.556	0.105
*CDH*13 (methylated)	0.997	0.948–1.048	0.903			
*CDKN1C* (methylated)	1.041	0.982–1.104	0.178			
*CDKN2A* (methylated)	1.281	1.169–1.404	< 0.001	1.018	0.623–1.664	0.943
*ESR1* (methylated)	1.20	1.108–1.303	< 0.001	1.005	0.684–1.475	0.981
*GSTP* (methylated)	1.126	1.065–1.19	< 0.001	1.18	1.008–1.40	0.02
*HIC1* (methylated)	1.110	1.061–1.162	< 0.001	0.845	0.644–1.109	0.225
*MGMT* (methylated)	1.184	1.107–1.268	< 0.001	1.026	0.861–1.223	0.774
*PRDM2* (methylated)	1.177	1.088–1.272	< 0.001	0.861	0.686–1.079	0.194
*PTEN* (methylated)	1.207	1.12–1.299	< 0.001	1.089	0.859–1.379	0.482
*PTGS2* (methylated)	0.888	0.845–0.933	< 0.001	0.978	0.834–1.147	0.787
*PYCARD* (methylated)	1.15	1.082–1.225	< 0.001	0.986	0.719–1.352	0.929
*RASSF1* (methylated)	1.138	1.078–1.201	< 0.001	1.196	0.959–1.490	0.112
SFN (methylated)	1.033	0.968–1.102	0.33			
*SLIT2* (methylated)	0.979	0.947–1.011	0.20			
*THBS1* (methylated)	1.17	1.092–1.256	< 0.001	0.936	0.640–1.367	0.731
*TNFRSF10C* (methylated)	0.948	0.923–0.974	< 0.001	1.005	0.948–1.065	0.879
*TP73* (methylated)	1.089	1.048–1.13	< 0.001	1.061	0.954–1.181	0.272
Non-TNBC (n = 50)						
*ADAM23* (methylated)	0.995	0.833–1.202	0.995			
*BRCA1* (methylated)	1.080	1.044–1.117	< 0.001	1.659	0.597–4.6411	0.332
*CCNA1* (methylated)	0.999	0.893–1.118	0.988			
*CCND2* (methylated)	0.940	0.837–1.055	0.294			
*CDH1* (methylated)	1.146	1.076–1.219	< 0.001	1.123	0.230–5.492	0.886
*CDH*13 (methylated)	0.957	0.862–1.063	0.413			
*CDKN1C* (methylated)	1.020	0.946–1.100	0.605			
*CDKN2A* (methylated)	1.408	1.205–1.645	< 0.001	5.790	0.379–88.564	0.207
*ESR1* (methylated)	1.113	1.064–1.164	< 0.001	0.596	0.099–3.586	0.572
*GSTP* (methylated)	1.150	1.085–1.219	< 0.001	0.756	0.044–13.019	0.847
*HIC1* (methylated)	1.107	1.047–1.171	< 0.001	1.452	0.470–4.48	0.517
*MGMT* (methylated)	1.257	1.125–1.405	< 0.001	0.176	0.007–4.504	0.294
*PRDM2* (methylated)	1.429	1.148–1.777	< 0.001	2.094	0.185–23.757	0.551
*PTEN* (methylated)	1.223	1.127–1.328	< 0.001	1.229	0.086–17.625	0.879
*PTGS2* (methylated)	0.883	0.840–0.928	< 0.001	0.562	0.097–3.241	0.519
*PYCARD* (methylated)	1.143	1.079–1.210	< 0.001	0.840	0.029–24.565	0.919
*RASSF1* (methylated)	1.252	1.069–1.465	< 0.001	2.517	0.575–11.024	0.220
SFN (methylated)	1.017	0.937–1.104	0.683			
*SLIT2* (methylated)	0.936	0.832–1.054	0.276			
*THBS1* (methylated)	1.066	1.030–1.102	< 0.001	0.501	0.155–1.619	0.248
*TNFRSF10C* (methylated)	0.969	0.938–1.001	0.059			
*TP73* (methylated)	1.073	1.018–1.131	< 0.001	1.118	0.365–3.421	0.845

Regarding the non-TNBC patients, worse DFS was markedly found in patients with hypermethylation levels of several genes including those involved in cell cycle regulation and apoptosis [*CDKN2A* (HR = 1.408, *p* < 0.001), *RASSF1* (HR = 1.252, *p* < 0.001), *PYCARD* (HR = 1.143, *p* < 0.001)], genes involved in angiogenesis and metastasis [*CDH1* (HR = 1.146, *p* =  < 0.001), *THBS1* (HR = 1.066, *p* < 0.001)], tumor suppressor genes including [*HIC1* (HR = 1.107, *p* < 0.001), *PTEN* (HR = 1.223, *p* < 0.001), *TP73* (HR = 1.073, *p* < 0.001)], DNA repair genes [*BRCA1* (HR = 1.080, *p* < 0.001), *MGMT* (HR = 1.257, *p* < 0.001)], histone methylation genes [*GSTP* (HR = 1.150, *p* < 0.001), *PRDM2* (HR = 1.429, *p* < 0.001)], and that coded for estrogen receptor [*ESR1* (HR = 1.113, *p* < 0.001)].

In contrast, *PTGS2* hypermethylation associated significantly with improved DFS [HR = 0.883, *p* < 0.001] in patients with non-TNBC.

Multivariate survival analysis did not identify the methylation status of any of the studied genes as an independent predictor of DFS in the non-TNBC cohort (Table 3).

### Association of gene methylation signature with the OS in BC, TNBC, and non-TNBC patients

In the entire cohort of BC patients, several genes demonstrated significant associations between their hypermethylation and the survival outcomes. These genes included *CDKN2A* (HR = 1.050, *p* = 0.028), *RASSF1* (HR = 1.037, *p* = 0.002), and *PYCARD* (HR = 1.054, *p* = 0.004) which hve important roles in cell cycle regulation and apoptosis. In addition to *THBS1* (HR = 1.031, *p* = 0.017) which concerned with angiogenesis and metastasis as. Similarly, there was a significant association between shorter OS and high methylation levels of tumor suppressor genes including [*HIC1* (HR = 1.044, *p* < 0.001), *PTEN* (HR = 1.032, *p* = 0.023)], DNA repair genes [*BRCA1* (HR = 1.02, *p* = 0.013), *MGMT* (HR = 1.035, *p* = 0.019)], and histone methylation genes [*GSTP* (HR = 1.027, *p* = 0.033)].

Conversely, *PTGS*2 methylation was associated with better OS [HR = 0.961, *p* = 0.006).

Finally, the methylation of *ADAM23*, *CCNA1, CCND2, CDH1, CDH13,* CDKN1C, *ESR1*, *PRDM2*, *SFN*, *SLIT2, TNFRSF10C,* and *TP73* did not show any significant associations with OS rate in BC patients.

The multivariate Cox regression analysis showed that there was no significant impact of the assessed gene methylation on the OS rate of the BC patients (Supp. 2).

Regarding the TNBC subgroup, higher methylation of several genes exhibited a strong association with poor OS rate in TNBC cohort. These genes included those elaborate in cell cycle regulation and apoptosis [*CDKN2A* (HR = 1.151, *p* = 0.005), *RASSF1* (HR = 1.108, *p* = 0.004), *PYCARD* (HR = 1.090, *p* = 0.011)], genes involved in angiogenesis and metastasis [*CDH1* (HR = 1.089, *p* = 0.014), *THBS1* (HR = 1.124, *p* < 0.001)], and tumor suppressor genes including [*HIC1* (HR = 1.057, *p* = 0.012), *PTEN* (HR = 1.083, *p* = 0.016), *TP73* (HR = 1.045, *p* = 0.027)]. Similarly, there was a significant association between poor OS rate of the TNBC patients and hypermethylation of DNA repair genes [*BRCA1* (HR = 1.071, *p* = 0.016), *MGMT* (HR = 1.102, *p* = 0.003)], histone methylation genes [*GSTP* (HR = 1.109, *p* = 0.009), *PRDM2* (HR = 1.105, *p* = 0.045)], and that coded for estrogen receptor [*ESR1* (HR = 1.127, *p* < 0.009)].

On the contrary, *PTGS*2 [HR = 0.933, *p* = 0.008] and *TNFRSF10C* [HR = 0.972, *p* = 0.04] methylation status associated significantly with better OS rate in the TNBC patients.

Finally, the methylation of *ADAM23*, *CCNA1, CCND2*, *CDH13*, *CDKN1C*, *SFN*, and *SLIT2* did not show any significant associations with the TNBC group.

Multivariate survival analysis showed that methylation status of *PTEN* [HR = 1.340, *p* = 0.030] was an independent predictor of OS in the TNBC cohort.

In non-TNBC patients, the findings showed no significant associations were observed between the methylation levels of the studied genes and OS rate of the non-TNBC patients (Table 4).

**Table 4:** Cox regression analysis for time to death (overall survival) in overall BC, TNBC and non-TNBC patients.

	Univariate analysis (OS)			Multivariate analysis (OS)		
	HR	95% CI	*p* value	HR	95% CI	*p* value
TNBC (n = 40)						
*ADAM23* (methylated)	0.951	0.896–1.009	0.094			
*BRCA1* (methylated)	1.071	1.031–1.132	0.016	1.050	0.858–1.287	0.634
*CCNA1* (methylated)	1.003	0.953–1.054	0.922			
*CCND2* (methylated)	1.006	0.934–1.084	0.871			
*CDH1* (methylated)	1.089	1.018–1.166	0.014	1.070	0.852–1.342	0.561
*CDH*13 (methylated)	1.011	0.945–1.082	0.747			
*CDKN1C* (methylated)	1.075	0.987–1.170	0.096			
*CDKN2A* (methylated)	1.151	1.044–1.296	0.005	0.917	0.557–1.508	0.732
*ESR1* (methylated)	1.127	1.031–1.233	0.009	0.874	0.634–1.205	0.410
*GSTP* (methylated)	1.109	1.026–1.199	0.009	1.047	0.868–1.264	0.630
*HIC1* (methylated)	1.057	1.012—1.104	0.012	1.027	0.845–1.248	0.791
*MGMT* (methylated)	1.102	1.033–1.176	0.003	1.225	0.948–1.583	0.120
*PRDM2* (methylated)	1.105	1.002–1.218	0.045	1.088	0.889–1.333	0.413
*PTEN* (methylated)	1.083	1.015–1.156	0.016	1.340	1.02–1.748	0.030
*PTGS2* (methylated)	0.933	0.887–0.982	0.008	0.988	0.837–1.165	0.884
*PYCARD* (methylated)	1.090	1.020–1.164	0.011	0.947	0.768–1.167	0.610
*RASSF1* (methylated)	1.108	1.034–1.188	0.004	1.117	0.953–1.310	0.172
SFN (methylated)	1.027	0.941–1.121	0.55			
*SLIT2* (methylated)	0.971	0.929–1.014	0.178			
*THBS1* (methylated)	1.124	1.038–1.218	< 0.001	1.211	0.952–1.540	0.119
*TNFRSF10C* (methylated)	0.972	0.947–0.999	0.04	1.055	0.988–1.126	0.109
*TP73* (methylated)	1.045	1.005–1.087	0.027	0.995	0.913–1.085	0.910
Non-TNBC (n = 50)						
*ADAM23* (methylated)	0.987	0.833–1.170	0.884			
*BRCA1* (methylated)	1.022	0.989–1.056	0.199			
*CCNA1* (methylated)	1.029	0.916–1.158	0.627			
*CCND2* (methylated)	0.939	0.844–1.044	0.243			
*CDH1* (methylated)	0.998	0.957–1.042	0.942			
*CDH*13 (methylated)	0.909	0.817–1.012	0.082			
*CDKN1C* (methylated)	0.983	0.906–1.066	0.677			
*CDKN2A* (methylated)	0.995	0.910–1.089	0.919			
*ESR1* (methylated)	1.009	0.967–1.053	0.672			
*GSTP* (methylated)	1.018	0.962–1.076	0.542			
*HIC1* (methylated)	1.033	1.000–1.068	0.052			
*MGMT* (methylated)	0.996	0.931–1.066	0.913			
*PRDM2* (methylated)	0.973	0.866–1.093	0.645			
*PTEN* (methylated)	1.019	0.949–1.093	0.608			
*PTGS2* (methylated)	0.878	0.947–1.047	0.878			
*PYCARD* (methylated)	1.014	0.957–1.074	0.643			
*RASSF1* (methylated)	1.020	0.981–1.060	0.317			
SFN (methylated)	1.022	0.941–1.110	0.605			
*SLIT2* (methylated)	1.007	0.903–1.123	0.898			
*THBS1* (methylated)	0.994	0.951–1.038	0.786			
*TNFRSF10C* (methylated)	1.027	0.988–1.068	0.179			
*TP73* (methylated)	0.953	0.905–1.004	0.071			

### Principal component analysis (PCA) of gene methylation profiles

A PCA was conducted to assess the variability in the methylation status of selected genes in TNBC and non-TNBC samples. The PCA plot (Fig. 1B**)** demonstrated a clear separation between the two groups. Principal components 1 and 2 explained 49.5% and 12.4% of the total variance, respectively, indicating that the selected methylation markers contributed significantly to distinguishing TNBC from non-TNBC samples. Samples clustered distinctly along the first principal component, which accounted for most of the variance, with TNBC samples predominantly positioned on one side of the plot and non-TNBC samples on the other. This separation suggests that the methylation patterns in the selected genes are differentially expressed between the TNBC and non-TNBC groups, potentially serving as robust biomarkers for classification.

### Heatmap visualization of methylated genes

**Fig. 2:**
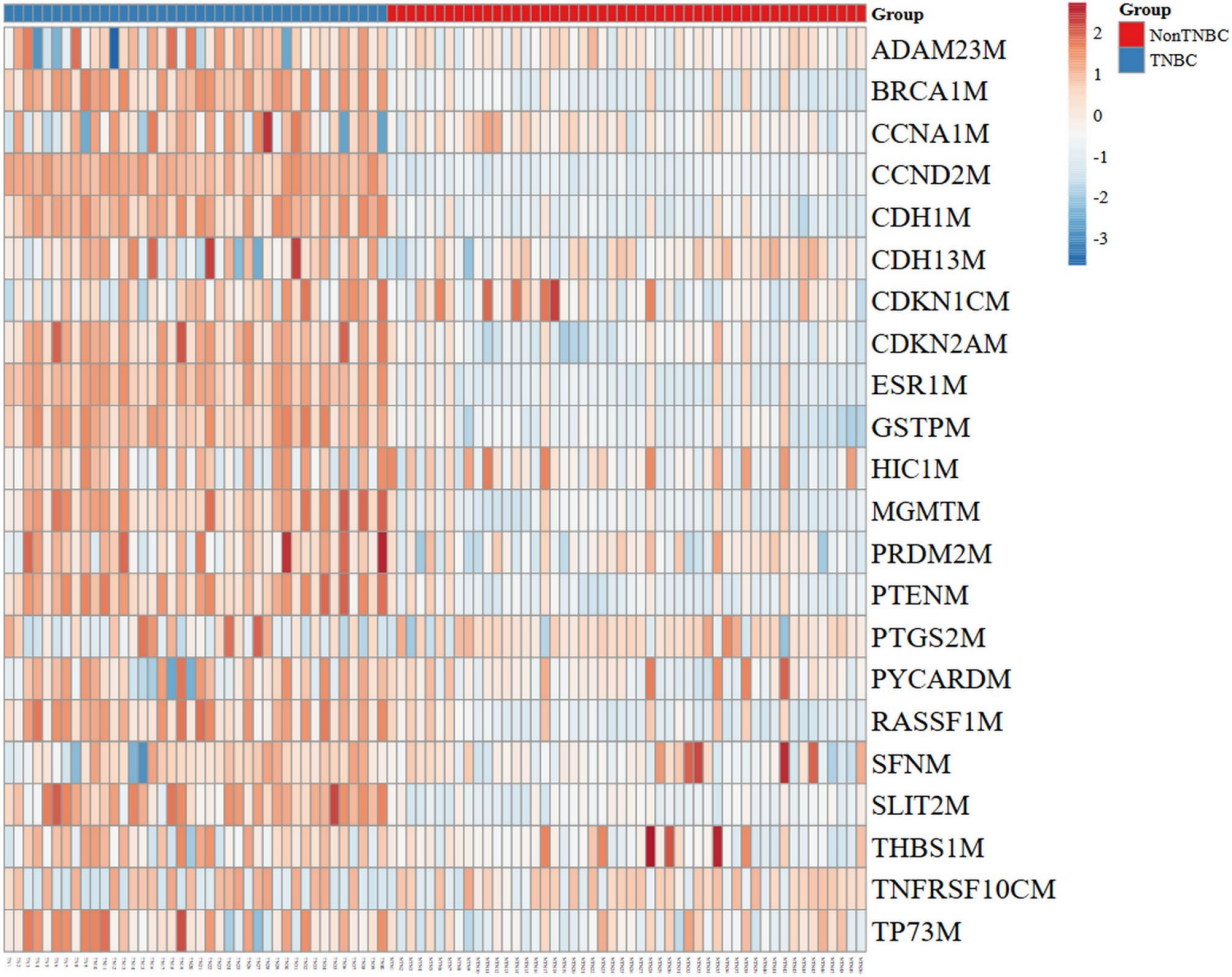
Heatmap diagram displaying the methylation levels of genes across the TNBC and non-TNBC groups.

A hierarchical clustering heatmap was generated using the methylation data (Fig. 2). The heatmap displayed the methylation levels of genes across the TNBC and non-TNBC samples, with samples grouped based on their methylation profiles. The heatmap clearly distinguished two major clusters corresponding to TNBC and non-TNBC groups. TNBC samples exhibited a distinct methylation pattern with hypermethylation in genes such as *CCND2, CDNK2A,* and *RASSF1***,** which involved in cell cycle regulation and apoptosis*, CDH1* which concerned with angiogenesis and metastasis, *BRCA1* and *MGMT* that have a role in DNA repair mechanism*, GSTP* which involved in histone methylation, tumor suppressor genes including *PTEN* and *SLIT2*, and finally the estrogen expression gene *ESR1.* While non-TNBC samples showed hypomethylation in the same previously mentioned genes. The hierarchical clustering dendrogram indicated tight clustering of TNBC samples together, suggesting that these methylation markers are consistently altered in this subtype of breast cancer based on their epigenetic profiles,

### Methylation profiles and clinical features in predicating BC subtypes

A Random Forest (RF) model was applied to evaluate the importance of various clinical and gene methylation features in predicting TNBC and non-TNBC subtypes. To ensure robust model performance and minimize overfitting, several strategies were implemented. The model utilized a dataset split into 80% training and 20% test sets, and a tenfold cross-validation approach was employed to evaluate performance across different splits. Additionally, the top-ranked features identified by importance scores were used to train the model, reducing the risk of overfitting by focusing on the most relevant predictors.

**Fig. 3:**
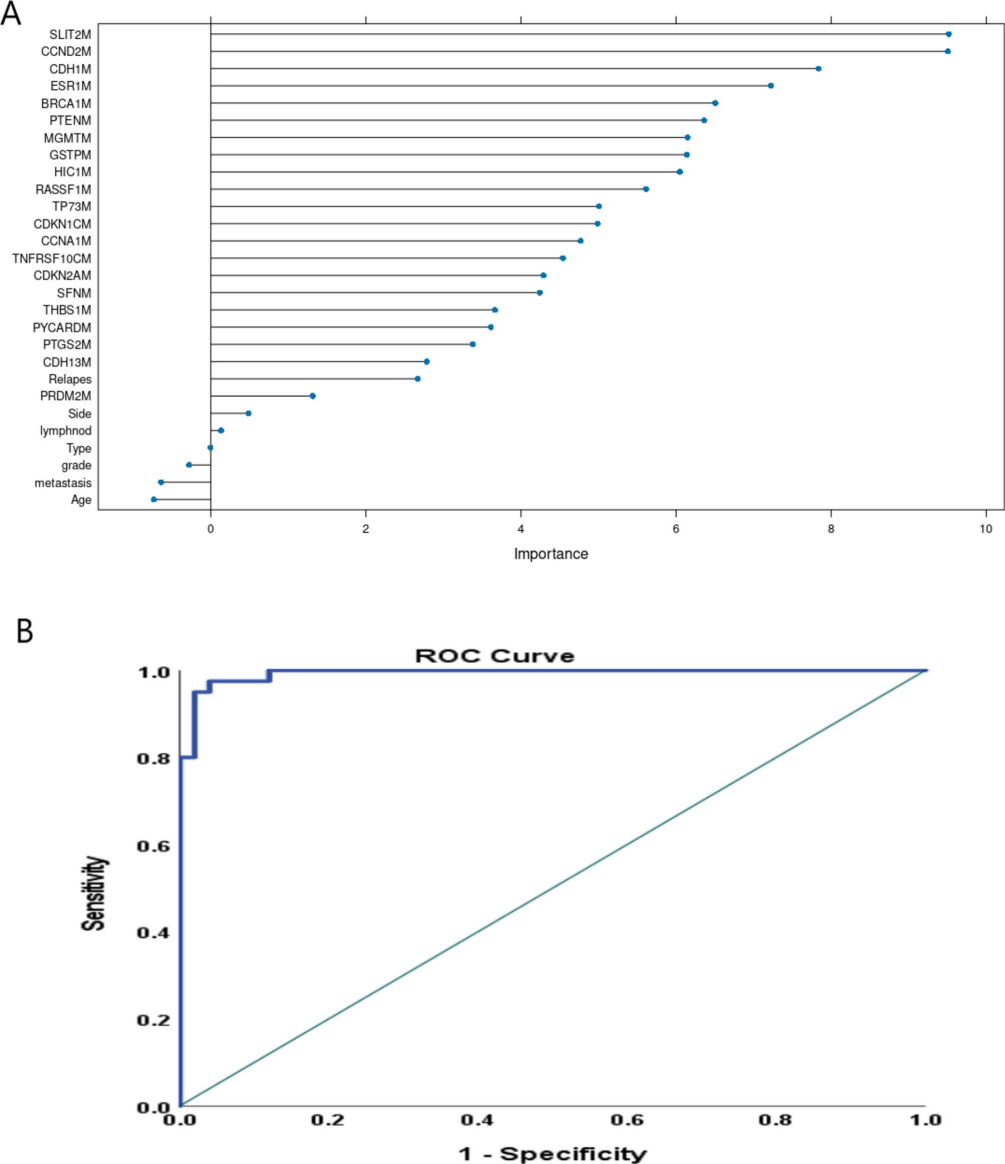
(**A**) The Model performance analysis showing several key methylation markers with their importance scores. (**B**) The ROC curve analysis to assess the performance for prediction of TNBC against non-TNBC patients.

As shown in Fig. 3A, the clinical variables had a minimal impact on the model’s performance. While age was a moderate predictor, relapse status showed a stronger association with BC subtype classification. The RF model identified key methylation markers with high importance scores, including SLIT2 (9.517), CCND2 (9.505), CDH1 (7.838), ESR1 (7.226), BRCA1 (6.505), PTEN (6.361), MGMT (6.148), and GSTP (6.143). Other important predictors included relapse status (a strong clinical predictor) and genes such as HIC1 (6.048), RASSF1 (5.613), and TP73 (5.006). These results highlight the critical role of epigenetic alterations in classifying BC subtypes. To further prevent overfitting, hyperparameter optimization was employed, ensuring the model’s generalizability.

Model performance was evaluated through ROC curve analysis, which demonstrated robust predictive capability for TNBC classification, with 95% sensitivity, 98% specificity, 97.44% positive predictive value, 94.23% negative predictive value and an AUC of 0.993 (Fig. 3B). These findings confirm the robustness of the RF model and its ability to identify key methylation-based predictors for BC subtype classification.

### Correlation among gene methylation in overall cohort, TNBC, non-TNBC groups

**Fig. 4:**
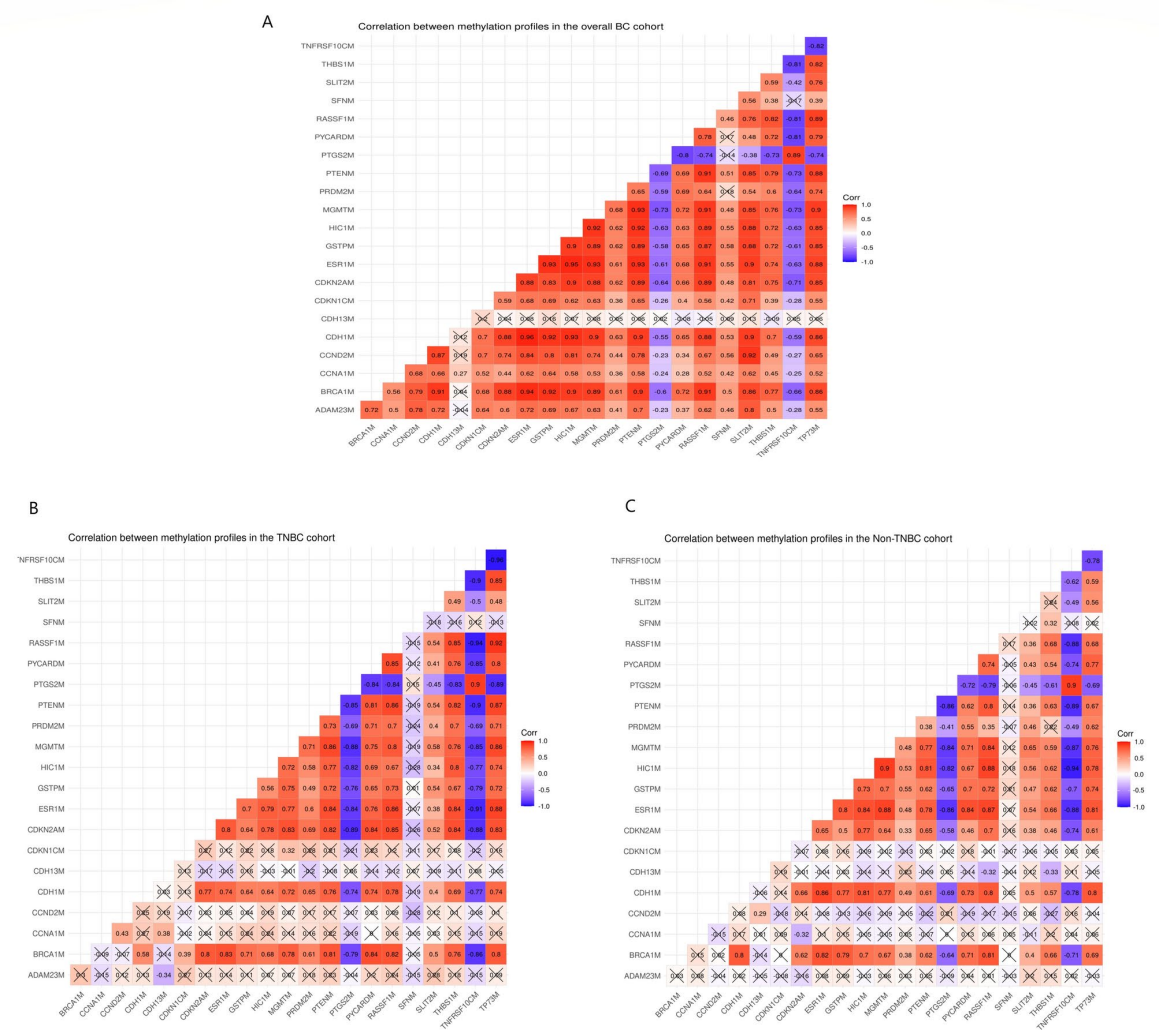
Correlation among the assessed genes in (**A**) overall breast cancer patients, (**B**) TNBC, and (C) non-TNBC patients.

Correlation analysis was performed among the assessed methylated genes and revealed positive correlations among most of the assessed genes except for *PTGS2* and *TNFRSF10C* which were inversely correlated with most of the methylated genes including *BRCA1*, *CDH1, CDKN2A*, *ESR1*, *GSTP1*, *HIC1, MGMT, PRDM2, PTEN PTGS2, PYCARD,* and *THBS1* (Fig. 4).

## Discussion

The TNBC is a major global health challenge, predominantly affecting premenopausal women, with nearly 50% of patients experiencing relapse within the first 3–5 years^24^. Despite current therapies, the median OS rate is only 10.2 months^25^. Epigenetic alterations, especially DNA promotor methylation, play a critical role in gene expression, molecular subtyping, prognosis, and clinical outcome of BC patients^26^.

The present study identified notable variations in the methylation status of several genes between TNBC and non-TNBC patients. Genes that were differentially hypermethylated in TNBC patients compared to those with non-TNBC were included in different vital pathways such as tumor suppressor genes (*SLIT2*, *PTEN*, *TP73*); cell cycle regulation and apoptosis (*CCND2, CDKN2A, RASSF1*), which control cell proliferation; cell adhesion and invasion mediators (*CDH1*), which influence epithelial-mesenchymal transition (EMT) and metastasis; DNA repair and genomic stability genes (*BRCA1*, *MGMT*), whose silencing may impair DNA repair mechanisms; histone methylation genes (*GSTP*, *PRDM2*), and finally, hormone receptor related genes (*ESR1*), whose methylation may contribute to hormone independence and endocrine therapy resistance. These findings highlight the distinct methylation landscape in TNBC pathogenesis and point toward potential key players in its molecular etiology. In consistent with these results, Guo et al., demonstrated 114 genes that were differentially methylated with altered expression in TNBC patients. These genes could be potential molecular targets for early detection and treatment of this type of BC^27^. Similarly, other studies found a distinct methylation pattern that can identify and early detect TNBC subtype^28–30^. Moreover, Manoochehri et al., proposed that this methylation pattern could be detected in in the plasma circulation cell-free DNA of those patients^29^. Consistently, many series reported a significant differential hypermethylation of different genes including *CDH1, ESR1*, and *MGMT* in TNBC patients^31–34^. Branham et al., also found that *CDKN2B* and MGMT methylation were significantly linked to TNBC, however they reported that *GSTP1* was not methylated in TNBC subtype^35^. These data highlighted the critical role of the promotor methylation in the pathogenesis of TNBC. On the other hand, no significant methylation differences were observed between TNBC and non-TNBC samples for genes such as *ADAM23, CCNA1, CDH13, CDKN1C, HIC1, PTGS2, PYCARD, SFN, THBS1,* and *TNFRSF10C* indicating that these genes may not contribute significantly to the methylation profile distinguishing TNBC from non-TNBC cases.

As revealed with PCA and Heatmap visualization, TNBC samples exhibited a distinct methylation pattern with hypermethylation in genes concerned with cell cycle regulation and apoptosis (*CCND2, CDKN2A, RASSF1*), angiogenesis and metastasis (*CDH1*), tumor suppressor genes (*SLIT2*, *PTEN*), DNA repair genes (*BRCA1*, *MGMT*), histone methylation genes (*GSTP*) and that coded for estrogen receptor (*ESR1*), while non-TNBC samples showed hypomethylation in the same genes.

The Random Forest model identified key methylation markers as critical predictors for distinguishing TNBC from non-TNBC subtypes, with genes such as *SLIT2, CCND2, CDH1, ESR1, BRCA1, PTEN, MGMT*, and *GSTP* demonstrating the highest importance scores. The strong association of these genes with TNBC underscores their pivotal role in the epigenetic landscape of BC and highlights their potential utility as biomarkers for subtype classification. Notably, the inclusion of these top-ranked genes in the predictive model significantly enhanced its ability to classify TNBC with high sensitivity (95%) and specificity (98%), achieving an AUC of 0.993. This underscores the biological and clinical relevance of these methylation markers as key contributors to TNBC stratification. Moreover, the prioritization of these features highlighted their potential as actionable targets for therapeutic intervention or as candidates for further validation in prospective studies. The model also highlighted relapse status as an important clinical predictor, underscoring its potential utility in guiding clinical decisions.

Regarding the impact of the hypermethylation epigenetic changes on the prognosis of the TNBC patients in terms of both DFS and OS outcomes. The current data demonstrated that hypermethylation of *BRCA1, MGMT* (DNA repair genes)*, CDKN2A, PYCARDM, RASSF1M* (cell cycle regulation and apoptosis), *CDH1, THBS1* (angiogenesis and metastasis)*, ESR1* (estrogen expression)*, HIC1*, *PTEN, TP73* (tumor suppressor genes), *GSTP*, and *PRDM2* (histone methylation genes) associated significantly with increased risk of worse OS and DFS rates in TNBC cohort. Meanwhile, *CCNA1* and *CDH1* (genes involved in cell cycle regulation and angiogenesis; respectively) demonstrated significant associations with poor DFS but did not show significant relationships with OS, indicating that their methylation levels primarily influence progression risk rather than overall survival. These findings are consistent with that reported by many studies identified differentially methylated regions (DMRs) that are significantly related to inferior outcomes and poor OS in TNBC patients^28,29,36^. Similarly, Yamashita et al., found that TNBC patients with *BRCA1* promoter methylation showed reduced OS and poor prognosis^37^. However, Jank et al. proposed that *MGMT* methylation status did not associate significantly with the DFS or OS rates in TNBC patients^38^. This discrepancy may owe to the fact that the study was performed on TNBC patients who were treated in the neoadjuvant GeparSixto trial^39^. While the current study was conducted on samples of untreated newly diagnosed patients. Therefore, chemotherapeutic agents may have an impact on the function of the gene.

In contrast, *PTGS2* and *TNFRSF10C* methylation were associated with better DFS and OS rates in TNBC patients, suggesting a protective role in reducing both disease recurrence/progression and mortality TNBC. Interestingly, PTGS2 methylation associated also with improved DFS in non-TNBC group, which may be explained by its role in inflammation and angiogenesis through the prostaglandin synthesis pathway^39^. Given that PTGS2 (COX-2) overexpression is linked to increased tumor aggressiveness and therapy resistance, its hypermethylation and subsequent silencing may reduce tumor progression^40^.

Similarly, the *TNFRSF10C* is a decoy receptor for the TNF-related apoptosis-inducing ligand (*TRAIL*), that is involved in the *TRAIL-R* signaling pathway^41^. Though *TNFRSF10C* is considered a ligand for apoptosis in several cancers, it also can promote tumor development and progression through activation of the NF-κB signaling pathway, which may explain its paradoxical protective effect in this cohort^42,43^.

Moreover, the current results revealed that hypermethylation of genes involved in cell cycle regulation and apoptosis *(CDKN2A, PYCARD, RASSF1)*, angiogenesis and metastasis (*THBS1)*, DNA repair (*BRCA1*, *MGMT*), histone methylation (*GSTP*) and tumor suppressor genes (*PTEN*, *HIC1*) were significantly associated with worse DFS and OS rates in the whole group of BC patients. While *CCND2* (cell cycle regulation), *CDH1* (angiogenesis and metastasis)*, ESR1*(estrogen expression)*, PRDM2* (histone methylation), and *TP73* (tumor suppressor gene) hypermethylation showed significant associations with poor DFS but did not show significant relationships with OS in BC patients. These findings are in agreement with Yadav et al., who reported that promotor hypermethylation of *BRCA1* and RASSF1 were linked significantly to increased mortality and poor outcomes in Indian BC patients^44^. Sheng and his colleagues also reported that *ESR1* promotor hypermethylation associated significantly with low OS in BC patients^45^. Additionally, Zekri et al., demonstrated that *CDH1* and *CCND2* are significantly downregulated in BCSCs, that linked to the pathogenesis and aggressiveness of BC^46^.

Further analysis showed that the methylation status of the tumor suppressor gene *PTEN*, and that involved in cell cycle regulation *CCND2* were independent predictors of DFS in the overall BC patients. While hypermethylation of *BRCA1* and *GSTP* which have role in DNA repair and histone methylation, respectively, were independent predictors of DFS in the TNBC cohort. Meanwhile the methylation status of the tumor suppressor gene *PTEN* was an independent predictor of OS in this cohort. This data are consistent with that observed by Ramadan et al., that *PTEN* methylation is a significant biomarker for inferior PFS and OS in BC patients^47^. Similarly, El Abbass et al., reported that *PTEN* associated with poor OS in BC patients^48^. These findings suggested that methylation status of *CCND2, PTEN*, *BRCA1* and *GSTP* could serve as potential biomarkers for predicting survival outcomes in BC patients, especially in TNBC, where the associations were stronger and more consistent. Future studies are needed to further validate these biomarkers and explore their potential for guiding treatment strategies.

In conclusion, the current study provided evidence that differential methylation profile of key genes in TNBC underscores its unique epigenetic features and provides valuable insights into its underlying molecular mechanisms. Genes such as *BRCA1*, *CCND2*, *CDH1, ESR1*, *GSTP*, *RASSF1*, *SLIT2, MGMT*, and *PTEN* emerge as promising biomarkers for the diagnosis, prognosis, and therapeutic targeting in TNBC. These hypermethylated genes could be integrated into diagnostic assays to enable earlier detection and more accurate molecular subtyping of TNBC. Additionally, the identified biomarkers may guide the development of targeted therapies, particularly for patients with poor prognosis, by allowing for personalized treatment strategies that focus on epigenetic modifications. Future studies should further validate these biomarkers on a larger scale of patients, with a validation cohort to properly confirm the results. Additionally, clinical trials should be performed to assess their utility in improving patient outcomes and informing treatment decisions.

## Supplementary Information

Supplementary Information.

## Data Availability

The datasets used and/or analysed during the current study are available from the corresponding author on reasonable request along with email: mona.sayed@nci.cu.edu.eg.
