# Supplementary material for: Molecular profiling of breast cancer methylation pattern in triple negative versus non- triple negative breast cancer
Source: Sci Rep. 2025 Feb 26;15:6894. doi: 10.1038/s41598-025-90150-9 (PMC11865568; doi:10.1038/s41598-025-90150-9)
Supplement: Supplementary file 1 — Supplementary Information. [file 41598_2025_90150_MOESM1_ESM.docx]

**Spp. 1:** Cox regression analysis for time to progression/relapse (progression-free survival) in BC patients.

|  | **Univariate analysis (DFS)** | | | **Multivariate analysis (DFS)** | | |
| --- | --- | --- | --- | --- | --- | --- |
|  | **HR** | **95% CI** | ***p* value** | **HR** | **95% CI** | ***p* value** |
| **BC patients (n=90)** | | | |  |  |  |
| ***ADAM23*** (methylated) | 0.98 | 0.93-1.04 | 0.46 |  |  |  |
| ***BRCA1*** (methylated) | 1.064 | 1.041-1.087 | **<0.001** | 1.077 | 0.973-1.191 | 0.152 |
| ***CCNA1*** (methylated) | 0.96 | 0.92-1.004 | 0.08 |  |  |  |
| ***CCND2*** (methylated) | 1.019 | 1.006-1.033 | **0.005** | 1.194 | 1.08-1.31 | **<0.001** |
| ***CDH1*** (methylated) | 1.054 | 1.033-1.075 | **<0.001** | 1.089 | 0.974-1.217 | 0.136 |
| ***CDH*13** (methylated) | 0.997 | 0.95-1.048 | 0.91 |  |  |  |
| ***CDKN1C*** (methylated) | 1.041 | 0.99-1.089 | 0.07 |  |  |  |
| ***CDKN2A*** (methylated) | 1.19 | 1.13-1.25 | **<0.001** | 1.157 | 0.898-1.491 | 0.258 |
| ***ESR1*** (methylated) | 1.073 | 1.044-1.103 | **<0.001** | 0.942 | 0.845-1.051 | 0.284 |
| ***GSTP*** (methylated) | 1.078 | 1.049-1.107 | **<0.001** | 0.904 | 0.778-1.050 | 0.188 |
| ***HIC1*** (methylated) | 1.106 | 1.071-1.142 | **<0.001** | 1.014 | 0.944-1.090 | 0.695 |
| ***MGMT*** (methylated) | 1.122 | 1.084-1.161 | **<0.001** | 1.083 | 0.957-1.225 | 0.205 |
| ***PRDM2*** (methylated) | 1.21 | 1.14-1.289 | **<0.001** | 0.898 | 0.784-1.028 | 0.120 |
| ***PTEN*** (methylated) | 1.11 | 1.076-1.149 | **<0.001** | 1.190 | 1.040-1.363 | **0.012** |
| ***PTGS2*** (methylated) | 0.872 | 0.842-0.904 | **<0.001** | 0.982 | 0.890-1.082 | 0.710 |
| ***PYCARD*** (methylated) | 1.169 | 1.118-1.222 | **<0.001** | 0.970 | 0.839-1.121 | 0.680 |
| ***RASSF1*** (methylated) | 1.10 | 1.070-1.133 | **<0.001** | 1.075 | 0.915-1.262 | 0.380 |
| **SFN** (methylated) | 1.058 | 0.974-1.15 | 0.182 |  |  |  |
| ***SLIT2*** (methylated) | 1.016 | 0.99-1.039 | 0.19 |  |  |  |
| ***THBS1*** (methylated) | 1.081 | 1.06-1.107 | **<0.001** | 0.999 | 0.909-1.098 | 0.983 |
| ***TNFRSF10C*** (methylated) | 0.951 | 0.931-0.970 | **<0.001** | 1.011 | 0.978-1.044 | 0.533 |
| ***TP73*** (methylated) | 1.079 | 1.051-1.109 | **<0.001** | 1.004 | 0.953-1.058 | 0.868 |

**Supp. 2:** Cox regression analysis for time to death (overall survival) in BC patients.

|  | **Univariate analysis (OS)** | | | **Multivariate analysis (OS)** | | |
| --- | --- | --- | --- | --- | --- | --- |
|  | **HR** | **95% CI** | ***p* value** | **HR** | **95% CI** | ***p* value** |
| **BC patients (n=90)** | | | |  |  |  |
| ***ADAM23*** (methylated) | 0.95 | 0.89-1.01 | 0.10 |  |  |  |
| ***BRCA1*** (methylated) | 1.02 | 1.004-1.037 | **0.013** | 1.011 | 0.958-1.066 | 0.693 |
| ***CCNA1*** (methylated) | 1.012 | 0.963-1.063 | 0.64 |  |  |  |
| ***CCND2*** (methylated) | 1.007 | 0.992-1.022 | 0.39 |  |  |  |
| ***CDH1*** (methylated) | 1.015 | 1.00-1.030 | 0.06 |  |  |  |
| ***CDH*13** (methylated) | 0.984 | 0.928-1.044 | 0.60 |  |  |  |
| ***CDKN1C*** (methylated) | 1.030 | 0.98-1.08 | 0.26 |  |  |  |
| ***CDKN2A*** (methylated) | 1.050 | 1.005-1.097 | **0.028** | 0.953 | 0.845-1.076 | 0.438 |
| ***ESR1*** (methylated) | 1.018 | 1.000-1.036 | 0.052 |  |  |  |
| ***GSTP*** (methylated) | 1.027 | 1.002-1.052 | **0.033** | 0.989 | 0.922-1.061 | 0.757 |
| ***HIC1*** (methylated) | 1.044 | 1.018-1.070 | **<0.001** | 1.028 | 0.989-1.070 | 0.164 |
| ***MGMT*** (methylated) | 1.035 | 1.006-1.065 | **0.019** | 0.997 | 0.900-1.104 | 0.948 |
| ***PRDM2*** (methylated) | 1.062 | 0.989-1.141 | 0.098 |  |  |  |
| ***PTEN*** (methylated) | 1.032 | 1.004-1.06 | **0.023** | 0.972 | 0.883-1.070 | 0.564 |
| ***PTGS2*** (methylated) | 0.961 | 0.934-0.989 | **0.006** | 1.005 | 0.941-1.073 | 0.891 |
| ***PYCARD*** (methylated) | 1.054 | 1.017-1.093 | **0.004** | 1.013 | 0.942-1.089 | 0.722 |
| ***RASSF1*** (methylated) | 1.037 | 1.013-1.061 | **0.002** | 1.070 | 0.987-1.161 | 0.102 |
| **SFN** (methylated) | 1.028 | 0.971-1.089 | 0.342 |  |  |  |
| ***SLIT2*** (methylated) | 1.00 | 0.973-1.028 | 0.99 |  |  |  |
| ***THBS1*** (methylated) | 1.031 | 1.005-1.058 | **0.017** | 0.981 | 0.930-1.034 | 0.472 |
| ***TNFRSF10C*** (methylated) | 0.989 | 0.971-1.006 | 0.202 |  |  |  |
| ***TP73*** (methylated) | 1.015 | 0.988-1.042 | 0.29 |  |  |  |
